# Polymer Functionalization of Isolated Mitochondria for Cellular Transplantation and Metabolic Phenotype Alteration

**DOI:** 10.1002/advs.201700530

**Published:** 2018-01-03

**Authors:** Suhong Wu, Aijun Zhang, Shumin Li, Somik Chatterjee, Ruogu Qi, Victor Segura‐Ibarra, Mauro Ferrari, Anisha Gupte, Elvin Blanco, Dale J. Hamilton

**Affiliations:** ^1^ Department of Nanomedicine Houston Methodist Research Institute Houston TX 77030 USA; ^2^ Center for Bioenergetics Houston Methodist Research Institute Houston TX 77030 USA; ^3^ Department of Medicine Weill Cornell Medicine New York NY 10065 USA; ^4^ Department of Physiology Weill Cornell Medicine New York NY 10065 USA; ^5^ Division Endocrinology Diabetes, and Metabolism Department of Medicine Houston Methodist Hospital Houston TX 77030 USA

**Keywords:** bioenergetic switches, cancer, cardiac failure, mitochondrial transplantation, surface modification/decoration

## Abstract

Aberrant mitochondrial energy transfer underlies prevalent chronic health conditions, including cancer, cardiovascular, and neurodegenerative diseases. Mitochondrial transplantation represents an innovative strategy aimed at restoring favorable metabolic phenotypes in cells with dysfunctional energy metabolism. While promising, significant barriers to in vivo translation of this approach abound, including limited cellular uptake and recognition of mitochondria as foreign. The objective is to functionalize isolated mitochondria with a biocompatible polymer to enhance cellular transplantation and eventual in vivo applications. Herein, it is demonstrated that grafting of a polymer conjugate composed of dextran with triphenylphosphonium onto isolated mitochondria protects the organelles and facilitates cellular internalization compared with uncoated mitochondria. Importantly, mitochondrial transplantation into cancer and cardiovascular cells has profound effects on respiration, mediating a shift toward improved oxidative phosphorylation, and reduced glycolysis. These findings represent the first demonstration of polymer functionalization of isolated mitochondria, highlighting a viable strategy for enabling clinical applications of mitochondrial transplantation.

## 1 Introduction

Efficient free energy transfer is necessary for normal cellular function. The failure to properly regulate mitochondrial bioenergetics underlies a variety of chronic diseases. As an example, high‐energy requiring cancer cells exhibit a phenotypic transition from oxidative phosphorylation (OXPHOS) to aerobic glycolysis (i.e., the Warburg effect),^1^ a phenomenon that in turn facilitates the growth and spread of these cells.^2^ On the other hand, reduced phosphocreatine‐to‐adenosine triphosphate (ATP) ratios observed in failing hearts highlight the energy‐depleted nature of the organ.^3^ Moreover, mitochondrial dysfunction is a hallmark of neurological conditions such as Parkinson's^4^ and Alzheimer's^5^ disease. Thus, novel treatment strategies aimed at improving aberrant energy metabolism stand to significantly impact the management of a variety of challenging disorders.^6^

Mitochondrial cell‐to‐cell transfer in response to stress has been previously reported.^7^, ^8^, ^9^, ^10^ In a report by Spees et al., active mitochondrial transfer occurred between adult stem cells and somatic cells, resulting in rescue of aerobic respiration in mammalian cells.^11^ Astrocytes were reported to shed mitochondria via membrane vesicles,^7^ which were then effectively transferred into neurons in response to cerebral ischemia, enhancing cell survival signaling.^8^ Transfer of mitochondria from healthy pheochromocytoma (PC) cells to stressed PC cells via F‐actin‐based tunneling nanotubes reversed apoptosis in culture,^10^ while mitochondrial transfer from mesenchymal stromal cells to macrophages along tunneling nanotubes enhanced the phagocytic activity of the latter, leading to improved antimicrobial effects in acute respiratory distress syndrome.^9^ Mitochondrial transfer from bone marrow‐derived stromal cells to pulmonary alveoli led to a reduction in inflammatory injury,^12^ while a recent study demonstrated that mitochondria released from platelets can undergo internalization into islet β‐cells and enhance their proliferation.^13^ The benefits observed following cell‐to‐cell mitochondrial transfer provide a rationale for investigating allogeneic or autologous mitochondrial transplantation as a potential therapeutic strategy targeting aberrant energy metabolism.

Transplantation of isolated mitochondria in vivo proves challenging. Among the barriers to clinical translation are limited cellular entry and the recognition of the organelle as foreign by the mononuclear phagocyte system (MPS) upon administration.^14^ Our objective was to biocompatibilize isolated mitochondria through surface functionalization with the polymer dextran so as to enhance transplantation into metabolically compromised cells. Incorporation of natural polysaccharides (e.g., dextran) and hydrophilic polymers such as poly(ethylene glycol) on the surface of nanoparticles has proven advantageous for in vivo delivery.^15^ These advantages include prolonged circulation times through prevention of protein adsorption, recognition by resident macrophages of the MPS (i.e., enhanced “stealthiness”), and stabilization that prevents aggregation.^16^ Moreover, the versatility of dextran allows for addition of moieties that enable molecular imaging or active targeting,^17^ as well as incorporation of additional therapeutics for synergy. Herein, we fabricated a dextran‐triphenylphosphonium (TPP) (Dextran‐TPP) conjugate as a mitochondrial coating. We aimed to examine cellular association and internalization of polymer‐functionalized mitochondria in cancer and cardiac cells, as well as oxidative changes in the metabolic phenotype resulting from mitochondrial transplantation. Findings highlight the feasibility of grafting Dextran‐TPP onto the surface of isolated mitochondria, with coated mitochondria entering into a dormant state characterized by reduced respiratory control ratio (RCR) and a reduced LEAK state (i.e., oxygen consumption in the absence of OXPHOS), a phenomenon not observed in uncoated mitochondria. Dextran‐TPP functionalization facilitated cellular internalization compared with uncoated mitochondria in a time‐dependent fashion, after which transplanted mitochondria induced a metabolic shift from glycolysis to OXPHOS with an increase in the oxygen consumption rate/extracellular acidification rate (OCR/ECAR) ratio in breast cancer and cardiac cells. Our findings are significant in that they represent the first study that demonstrates enhanced cellular transplantation of mitochondria resulting from polymer functionalization of the organelle's surfaces, and the first to highlight the ensuing alteration in energy metabolism that can potentially impact a variety of diseases.

## 2 Results and Discussion

### 2.1 Dextran‐TPP Polymer Comprehensively Coated Isolated Mitochondria

**Figure 1:**
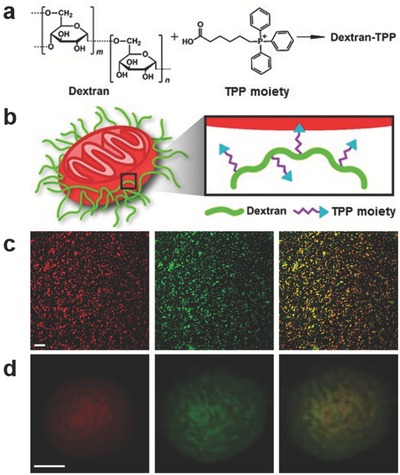
Mitochondria functionalized with a Dextran‐TPP polymer coating for cellular transplantation. a) Chemical structures of dextran and TPP moiety, constituent materials of the mitochondrial polymer coating. b) Schematic of Dextran‐TPP coating of mitochondria, highlighting TPP incorporation into the mitochondrion. c) Confocal microscopy of Dextran‐TPP‐coated mouse liver‐derived mitochondria. Isolated mitochondria were stained with MitoTracker Deep Red (red) and Dextran‐TPP was labeled with FITC (green). The scale bar in the images represents 20 µm. d) Magnification of a Dextran‐TPP‐coated mitochondrion examined via confocal microscopy. The scale bar represents 0.5 µm.

Our innovative strategy of functionalizing mitochondria with a polymer coating was meant to confer advantages for mitochondrial transplantation. Incorporation of hydrophilic polymers onto nanomaterial surfaces has long been associated with biocompatibility, enhanced stability, and longer retention times in vivo.^18^ Dextran was selected as the polymer coating based on its wide use in a number of biomedical applications,^19^ with advantages that include biocompatibility and the potential for further functionalization. To obtain stable association with mitochondrial membranes, dextran was conjugated with TPP (**Figure** **1**a), a lipophilic, cationic ligand with mitochondriotropic properties (Figure 1b).^20^ Dextran‐TPP conjugation proved successful, as demonstrated by ^1^H NMR (Figure S1, Supporting Information). Upon functionalization of mitochondria with the Dextran‐TPP polymer, coating of mitochondria was shown to be dependent on Dextran‐TPP:mitochondria weight ratio, with higher ratios (1.9:1 and 2.9:1) resulting in a more complete and comprehensive coating of isolated mitochondria than a lower ratio of 1.4:1 (Figure S2, Supporting Information). Upon mitochondrial respiration examination of mitochondria coated at different ratios, a concentration‐dependent decline in the pyruvate–malate state 3 rate was observed, and the oligomycin state 4 rate was elevated with the 2.9:1 concentration, indicating uncoupling or LEAK (Figure S3, Supporting Information). Consequently, for all subsequent experiments involving mitochondrial functionalization, a ratio of 1.9:1 Dextran‐TPP:mitochondria was used. Mitochondrial coating was further confirmed by zeta potential analysis, with findings demonstrating that the negative surface charge of the organelles (−44 mV) transitioned toward a much less negative charge following functionalization with Dextran‐TPP, reaching an average of −4 mV (Figure S4, Supporting Information). Confocal microscopy examination of coated mitochondria indeed demonstrated successful coating of mitochondria with the polymer. As can be seen in Figure 1c, a comprehensive coating of mitochondria was achieved, as demonstrated by colocalization of mitochondria stained with MitoTracker Red and fluorescein 5(6)‐isothiocyanate (FITC) (green) fluorescently‐labeled Dextran‐TPP, with successful polymer functionalization highly evident in the magnified image of a single coated mitochondrion (Figure 1d). Coating of isolated mitochondria by Dextran‐TPP conjugates is owed primarily to the large lipophilic surface area of the TPP cation, which is able to effectively amass within the negatively charged mitochondrial matrix.^21^

### 2.2 Polymer Functionalization of Mitochondria Resulted in a Dormant Respiratory State

**Figure 2:**
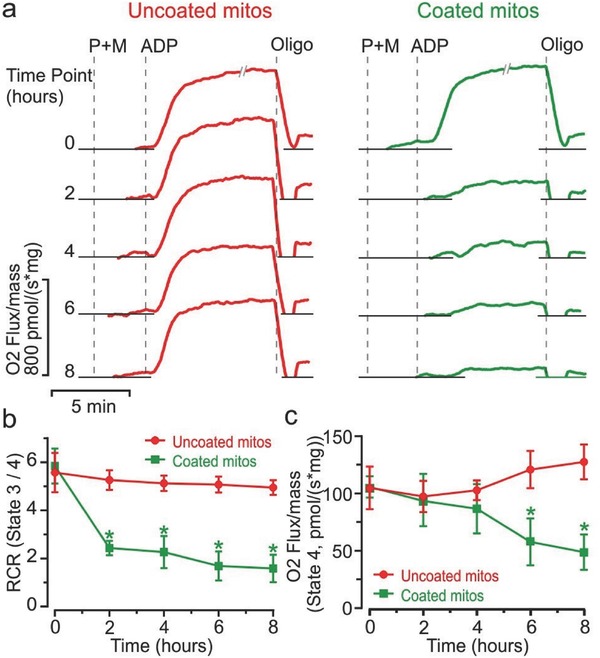
Functional assessment of Dextran‐TPP‐coated mitochondria isolated from mouse livers. a) Oxygen consumption as a function of time of uncoated and Dextran‐TPP‐coated mouse liver‐derived mitochondria in response to addition of pyruvate (P, 5 × 10^−3^ m), malate (M, 2 × 10^−3^ m), ADP (2 × 10^−3^ m), and oligomycin (Oligo, 4 × 10^−6^ m). The break in oxygen flux for the 0 h time point lasts 1.5 min. b) RCR (state 3/4) as a function of time, with state 3 being the response to ADP, and state 4 representing the response to oligomycin. c) Oxygen flux as a function of time following the addition of oligomycin. Results represent mean ± SEM (*P < 0.05 compared with uncoated mitos group).

Respiratory function of uncoated and coated mitochondria was assessed with Oroboros high‐resolution respirometry. There was no difference in the time 0 h oxygen flux response to adenosine diphosphate (ADP) after substrate addition (state 3) in uncoated controls (680 ± 45 pmol [s mg]^−1^) compared with precoated mitochondria (685 ± 76 pmol [s mg]^−1^) (**Figure** **2**a). At the 0 h time point, the Dextran‐TPP functionalization process was immediately initiated, and the respiratory function can be considered as basal level at this time. At the 2 h time point, the ADP response in coated mitochondria decreased to 153 ± 25 pmol (s mg)^−1^, a 78% decline compared with the uncoated group, which remained unchanged at the 2 h (683 ± 51 pmol [s mg]^−1^) and 8 h time points (656 ± 38 pmol [s mg]^−1^). Oxygen flux in uncoated controls at each time point showed no significant difference compared with the 0 h time point. In contrast, coated mitochondria at 8 h (63 ± 12 pmol [s mg]^−1^) remained significantly suppressed compared with the 0 h time point, but with no significant change compared with the 2 h time point (Figure 2a).

To evaluate the OXPHOS coupling efficiency, the RCR, defined as the ratio of state 3 (substrate and ADP) to state 4 respiration (oligomycin addition), was determined (Figure 2b). The RCR index decreased from 5.26 ± 0.20 at time 0 h to 2.44 ± 0.17 at 2 h after Dextran‐TPP coating. The RCR of uncoated mitochondria had no significant drop after 2 h (5.85 ± 0.42 compared with 5.57 ± 0.41 at the 0 h time point).

To examine if the RCR decline was due to loss of ADP‐activated state 3 respiration or increased LEAK respiration (state 4, oligomycin addition), we assessed oxygen flux rates after addition of oligomycin, a mitochondria complex V inhibitor that enhances membrane LEAK respiration (Figure 2c). At 4 h, there was no difference in LEAK respiration rates between coated and uncoated mitochondria. At 6 h, the LEAK rate in the coated group significantly decreased from 106 ± 9 pmol (s mg)^−1^ at time 0 h to 58 ± 29 pmol (s mg)^−1^. By comparison, a slight LEAK increase was observed in the uncoated group between time 0 h (105 ± 19 pmol [s mg]^−1^) and 6 h (121 ± 16 pmol [s mg]^−1^). The reduced RCR in the coated group was the result of reduced ADP state 3 respiration rather than increased LEAK, given that a reduced LEAK rate was observed in coated mitochondria compared with uncoated controls.

Dextran functionalization of mitochondria effectively protected respiratory function by placing isolated mitochondria in a state of metabolic dormancy. While RCRs of coated mitochondria were reduced compared with uncoated mitochondria, reduced oxygen LEAK rates reflect maintenance of OXPHOS integrity compared with uncoated mitochondria. While coated mitochondria were in a dormant state prior to transplantation, they recovered following cellular transplantation. Metabolic depression of coated mitochondria, a phenomenon similar to that observed in mitochondria isolated from animals in a hibernation state with reduced metabolism,^22^ may very well be due to limited substrate entry into mitochondria because of the dextran coating. It is important to note that this dormant state was not observed with uncoated mitochondria, suggesting a potential benefit for mitochondrial ex vivo preservation.

### 2.3 Dextran Coating of Isolated Mitochondria Facilitated Cellular Internalization

**Figure 3:**
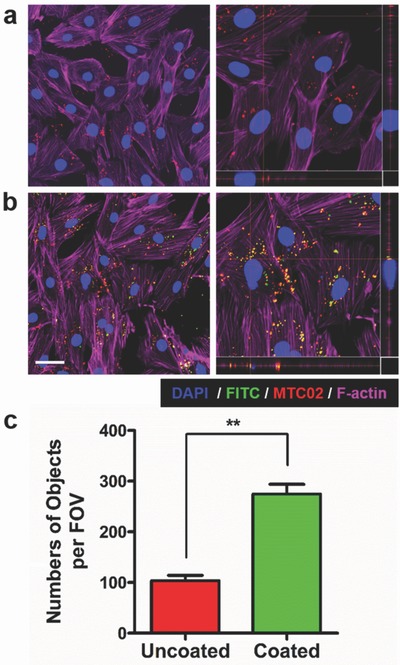
Uptake and intracellular localization of HeLa‐derived mitochondria in H9c2 cardiac myoblast cells. Confocal microscopy images of H9c2 heart myoblast cells incubated for 24 h with a) uncoated or b) Dextran‐TPP/FITC‐coated HeLa‐derived mitochondria (3 µg mitochondrial protein per 1.5 × 10^4^ cells). Nuclei were stained with DAPI (blue) and mitochondria coated with Dextran‐TPP/FITC (green). HeLa mitochondria were detected with anti‐human mitochondrial antibody (MTCO2) and anti‐mouse IgG antibody (red). F‐actin was stained with Alexa Fluor Phalloidin‐647 (purple). Images represent low magnification (left) and 2D images (right), with panels below and to the right of 2D images highlighting mitochondrial internalization. The scale bar represents 50 µm. c) Average number of internalized mitochondria, uncoated and Dextran‐TPP coated, per field of view in H9c2 heart myoblast cells. Results represent mean ± SEM (*P < 0.01 compared with uncoated mitos group).

The ability of coated mitochondria to undergo cellular entry was examined in H9c2 heart myoblast cells via confocal microscopy (**Figure** **3**). Rat H9c2 cells were chosen for ease of identification of HeLa cell‐derived mitochondria via fluorescence‐based immunohistochemistry, enabling differentiation of native versus transplanted human mitochondria. Figure S5a (Supporting Information) shows that a small degree of internalization of uncoated mitochondria occurred after 4 h incubation with H9c2 cells, with mitochondria mostly confined to extracellular spaces and cellular membranes. Mitochondria functionalized with Dextran‐TPP underwent a greater degree of uptake in H9c2 cells after 4 h compared with the uncoated group, albeit, with a large amount of mitochondria also found outside of cells at this time point (Figure S5b, Supporting Information). An approximate 3‐fold difference in internalization was observed between coated and uncoated mitochondria 4 h after incubation (Figure S5c, Supporting Information). After 24 h incubation, uncoated mitochondria underwent increased internalization within H9c2 cells compared with the 4 h time point (Figure 3a). However, mitochondrial uptake into cells after 24 h was substantially greater following functionalization with Dextran‐TPP (Figure 3b). Once more, an approximate 3‐fold difference in accumulation compared with the uncoated mitochondria group was observed at 24 h (Figure 3c). Similar findings were observed in a separate study performed in mouse L929 fibroblast cells, wherein HeLa‐derived mitochondria coated with Dextran‐TPP remained confined to extracellular spaces and cellular membranes at earlier time points (Figure S6, Supporting Information), but underwent substantial uptake by the 24 h time point (Figure S7, Supporting Information). It is important to note that an increase in uptake of coated mitochondria by L929 cells was observed compared with uncoated mitochondria at both time points.

The significant cellular internalization advantage provided by polymer functionalization can best be explained by the effect of the Dextran‐TPP coating on the surface of mitochondria. As we demonstrated, mitochondria have a highly negative charge that was substantially reduced following coating with the Dextran‐TPP conjugate. Cellular uptake of materials on the nano‐ and microscale is driven by charge, with positively charged materials undergoing higher rates of internalization due to attractive electrostatic interactions with the negatively charged cell membrane.^23^ Additionally, TPP is a hydrophobic cation,^24^ possessing a large lipophilic surface area that allows it to traverse phospholipid bilayers^21^ and promote efficient cellular uptake regardless of cell type through hydrophobic interactions with cell membrane components or via integration into cell membranes.^25^ Thus, the TPP associated with mitochondrial membranes may be coming into direct contact with cell membranes, resulting in increased cellular accumulation. Taken together, mitochondrial functionalization with Dextran‐TPP can enhance uptake into target cells.

**Figure 4:**
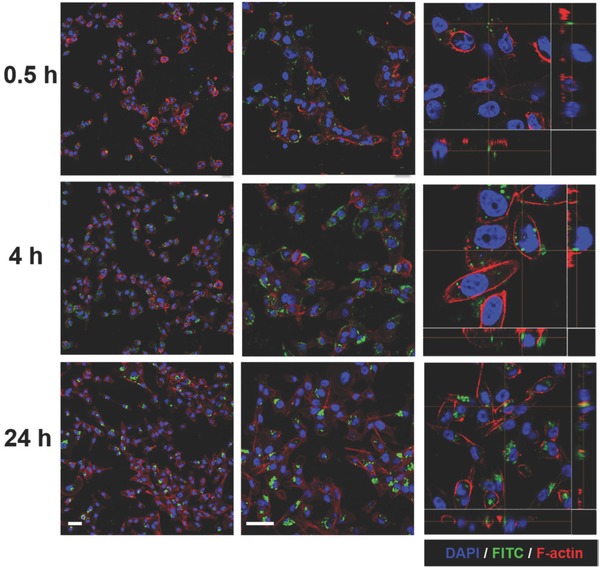
Mitochondrial transplantation into MDA‐MB‐231 breast cancer cells. Confocal microscopy images of MDA‐MB‐231 breast cancer cells incubated with Dextran‐TPP‐coated mouse liver‐derived mitochondria (3 µg mitochondrial protein per 1.5 × 10^4^ cells) at time points of 0.5, 4, and 24 h. Mitochondria coated with Dextran‐TPP/FITC appear as green, nuclei were stained with DAPI (blue), and F‐actin was stained with Alexa Fluor Phalloidin‐568 (red). Images represent low magnification (left), enlarged view (middle), and 2D images (right). Panels below and to the right of 2D images highlight mitochondrial internalization. The scale bar represents 20 µm.

**Figure 5:**
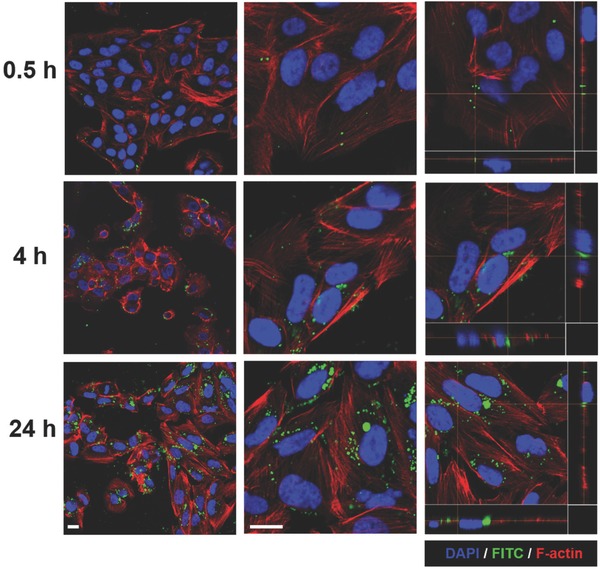
Mitochondrial transplantation into SUM‐159PT breast cancer cells. Confocal microscopy images of SUM‐159PT breast cancer cells incubated with Dextran‐TPP‐coated mouse liver‐derived mitochondria (3 µg mitochondrial protein per 1.5 × 10^4^ cells) at time points of 0.5, 4, and 24 h. Mitochondria coated with Dextran‐TPP/FITC appear as green, nuclei were stained with DAPI (blue), and F‐actin was stained with Alexa Fluor Phalloidin‐568 (red). Images represent low magnification (left), enlarged view (middle), and 2D images (right). Panels below and to the right of 2D images highlight mitochondrial internalization. The scale bar represents 20 µm.

Having successfully demonstrated increased cellular internalization of Dextran‐TPP‐functionalized mitochondria compared with uncoated mitochondria, the kinetics of cellular association and internalization was examined in human MDA‐MB‐231 and SUM‐159PT triple negative breast cancer (TNBC) cell lines. As can be observed in **Figure** **4**, mitochondrial internalization in MDA‐MB‐231 cells was time dependent. At early time points of 0.5 h, polymer‐coated mitochondria were found sparsely associated with cells. At time points of 4 h after incubation, mitochondria were mostly found around cell membranes. By 24 h, there was a substantial increase in mitochondrial uptake within cells (Figure 4). As is evident in the confocal micrographs, internalized mitochondria were found in the cell cytoplasm as early as 4 h after incubation. A similar pattern of time‐dependent internalization of polymer‐coated mitochondria was also observed in SUM‐159PT TNBC cells (**Figure** **5**).

### 2.4 Transplantation of Polymer‐Functionalized Mitochondria into Breast Cancer and Cardiac Cell Lines Resulted in Respiratory Changes

The benefits of compensatory endogenous mitochondrial transfer through extracellular spaces into metabolically compromised cells in a variety of prevalent acquired diseases provided the impetus for investigating mitochondrial transplantation as a viable treatment approach by our laboratory as well as by other groups.^26^ To determine whether transplantation of polymer‐functionalized mitochondria was capable of triggering a bioenergetic switch in cells of varying origin and phenotype, simultaneous OCR and ECAR were assessed. All cells, regardless of treatment, respired effectively with substrate and following sequential addition of inhibitors and uncouplers (Figure S8, Supporting Information). After assessing OCR and ECAR in eight cell lines, we selected two cancer cell lines and two cardiac cell types as representative targets for transplantation. We chose two breast cancer cell lines, MDA‐MB‐231 and SUM‐159PT, characterized by high aerobic glycolytic rates (Figure S8, Supporting Information). As representatives of cardiac cells, heart myoblast H9c2 cells and isolated cardiomyocytes (CMs) were selected. Cardiac cells exhibit a greater OXPHOS to glycolytic ratio than the cancer cell lines and were chosen to assess for the bioenergetic impact of transplanted mitochondria in cells that do not exhibit a dominant glycolytic pattern.

**Figure 6:**
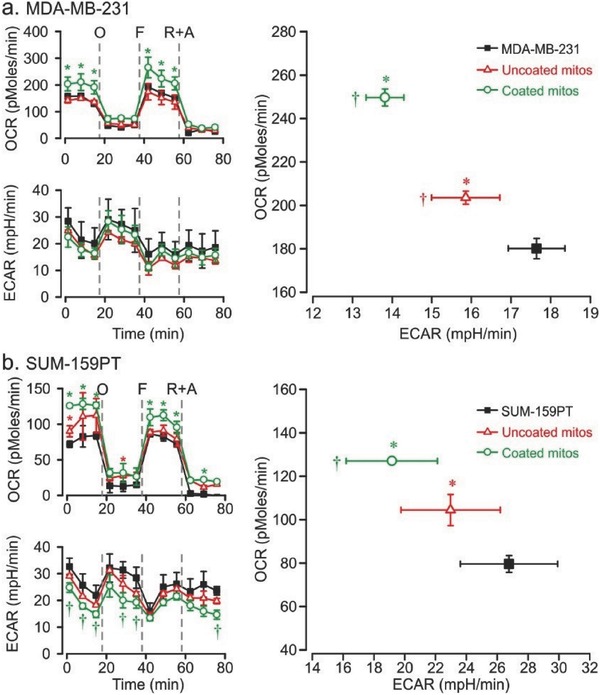
Mitochondrial oxygen respiration following transplantation of Dextran‐TPP‐coated mitochondria into human TNBC cells. OCR and ECAR response of a) MDA‐MB‐231 and b) SUM‐159PT breast cancer cells 24 h after transplantation of uncoated (red) and Dextran‐TPP‐coated (green) mouse liver‐derived mitochondria (10 µg mitochondrial protein per 1.5 × 10^4^ cells). OCR as a function of ECAR (right panel) is derived from basal measurements. Results represent mean ± SEM. (*P < 0.05 and ^†^ *P* < 0.05 compared with OCR and ECAR, respectively, in nontreated cells.) O: oligomycin; F: FCCP; R + A: rotenone + antimycin A.

We examined MDA‐MB‐231 cells alone and after addition of coated and uncoated mitochondria. As can be seen in **Figure** **6**a, MDA‐MB‐231 is a glycolysis dominated cell line. Interestingly, the basal OCR was significantly increased in the coated group (210.5 ± 30.7 pmol min^−1^) compared with nontreated MDA‐MB‐231 cells (158.7 ± 5.2 pmol min^−1^). The maximum OCR capacity (FCCP addition) also increased significantly in the coated group (265.4 ± 38.7 pmol min^−1^) compared with nontreated MDA‐MB‐231 cells (192.3 ± 6.75 pmol min^−1^). These findings indicate that addition of coated mitochondria accelerated the electron transfer rate to oxygen in the basal and uncoupled state. LEAK respiration and nonmitochondrial oxygen consumption (rotenone and antimycin A addition) did not change. We assessed the average basal OCR and ECAR prior to addition of oligomycin (Figure 6a, right panel) and found a significant shift from ECAR to OCR in both coated and uncoated groups, indicating a switch from a glycolytic to an oxidative phenotype. However, a greater shift was recorded in the coated group compared with the uncoated group, with both OCR and ECAR changing significantly (*p* < 0.05). The OCR of the nontreated MDA‐MB‐231 group was 180.2 ± 4.7 pmol min^−1^, while that of the uncoated and coated groups increased to 203.6 ± 3.0 and 249.7 ± 4.4 pmol min^−1^, respectively. The ECAR of the nontreated MDA‐MB‐231 group was 17.6 ± 0.7 mpH min^−1^, while that of the uncoated and coated groups decreased to 15.9 ± 0.9 and 13.8 ± 0.5 mpH min^−1^, respectively.

To confirm the bioenergetic switch observed in MDA‐MB‐231 cells, OCR and ECAR were examined in the SUM‐159PT TNBC cell line (Figure 6b; Figure S9, Supporting Information). OCR and ECAR changes after coated and uncoated mitochondrial transplantation were consistent with those observed in MDA‐MB‐231 cells. The basal SUM‐159PT OCR significantly increased in the coated group (126 ± 2.8 pmol min^−1^) compared with nontreated SUM‐159PT cells (71 ± 4.6 pmol min^−1^). The maximum OCR capacity increased in the coated group (110 ± 11.8 pmol min^−1^) compared with nontreated SUM‐159PT cells (86.4 ± 2.8 pmol min^−1^). Upon averaging of the OCR and ECAR, a significant shift from ECAR to OCR in both the coated and uncoated groups was indicative of a cell metabolism switch from a glycolytic to oxidative state. The coated group produced a greater shift than the uncoated group, with both OCR and ECAR changing significantly (*p* < 0.05). The OCR of nontreated SUM‐159PT cells was 80 ± 3.9 pmol min^−1^. After transplantation of coated mitochondria, OCR increased to 127 ± 2.8 pmol min^−1^ while the OCR value of cells treated with uncoated mitochondria was 104 ± 7.2 pmol min^−1^. The ECAR of nontreated SUM‐159PT cells was 26.8 ± 3.2 mpH min^−1^. This value subsequently decreased to 23 ± 3.2 and 19.2 ± 0.8 mpH min^−1^ following the addition of uncoated and coated mitochondria, respectively.

**Figure 7:**
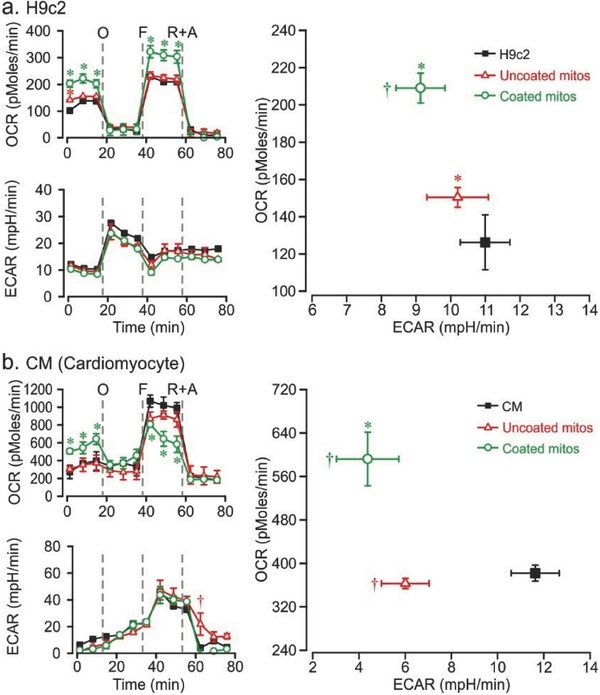
Mitochondrial oxygen respiration following transplantation of Dextran‐TPP‐coated mitochondria into cardiac cells. OCR and ECAR response of a) H9c2 cardiac myoblast cells and b) primary adult mouse CMs 24 h after transplantation of uncoated (red) and Dextran‐TPP‐coated (green) mouse liver‐derived mitochondria (10 µg mitochondrial protein per 1.5 × 10^4^ cells). OCR as a function of ECAR (right panel) is derived from basal measurements. Results represent mean ± SEM. (*P < 0.05 and ^†^ *P* < 0.05 compared with OCR and ECAR, respectively, in nontreated cells.) O: oligomycin; F: FCCP; R + A: rotenone + antimycin A.

To determine the effect of coated mitochondrial transplantation on cardiac cell bioenergetics, OCR and ECAR were examined in an H9c2 cell line and adult mouse CMs. Basal OCR significantly increased in the coated group (202.9 ± 13.3 pmol min^−1^) compared with nontreated H9c2 cells (102.1 ± 4.2 pmol min^−1^) (**Figure** **7**a; Figure S10, Supporting Information). The maximum OCR capacity (FCCP addition) also increased in the coated group (322.4 ± 22.6 pmol min^−1^) compared with nontreated H9c2 cells (229.3 ± 4.7 pmol min^−1^). A significant shift from ECAR to OCR occurred in the coated group (OCR 209.1 ± 8.0 pmol min^−1^, ECAR 9.1 ± 0.7 mpH min^−1^) compared with nontreated H9c2 cells (OCR 126.2 ± 14.8 pmol min^−1^, ECAR 11.0 ± 0.7 mpH min^−1^). The ECAR did not change significantly in the uncoated group, but the OCR significantly increased to 150.4 ± 5.5 pmol min^−1^. Minimal LEAK respiration occurred after the addition of oligomycin, and differences in mitochondrial oxygen consumption following addition of rotenone and antimycin A were not significant.

In CMs, basal OCR significantly increased in the coated group (505.3 ± 27.6 pmol min^−1^) compared with nontreated CMs (273.5 ± 75.0 pmol min^−1^) (Figure 7b). The maximum OCR capacity was lowest in the coated group (805.2 ± 23.7 pmol min^−1^) compared with nontreated CMs (1068.1 ± 67.9 pmol min^−1^). A significant shift from ECAR to OCR was observed in the coated group (OCR 592.2 ± 49.5 pmol min^−1^, ECAR 4.4 ± 1.3 mpH min^−1^) compared with nontreated CMs (OCR 382.0 ± 14.8 pmol min^−1^, ECAR 11.6 ± 1.0 mpH min^−1^). While the ECAR decreased in the uncoated group (6.0 ± 1.0 mpH min^−1^), the OCR did not change significantly.

The TNBC cell lines MDA‐MB‐231 and SUM‐159PT exhibited a baseline glycolytic profile. In contrast, high energy‐demanding CMs have an abundance of mitochondria, constituting 30–40% of the cellular volume.^27^ Consequently, 95% of the ATP is produced by OXPHOS and the remaining 5% by glycolysis.^28^ Thus, CMs are committed to OXPHOS and cannot efficiently balance between OXPHOS and glycolysis as efficiently as other cells. The acidification observed may result not only from glycolytic lactate release, but also from increased Kreb's cycle flux releasing CO_2_ and carbonic acid release. Therefore, the inverse relationship of ECAR to OXPHOS is not as closely linked in CMs as in MDA‐MB‐231, SUM‐159PT, or H9c2 cells, the latter of which demonstrates an ECAR increase in a compensatory response to OCR decrease.^29^

The considerable shift in bioenergetic phenotype following transplantation of polymer‐functionalized mitochondria is most likely the result of increased number of cellular mitochondria. Mitochondria functionalized with dextran showed a much higher accumulation than uncoated mitochondria, and thus, a greater shift toward increased OXPHOS. It is now well known that mitochondrial number and function change in response to cell stress and changes in phenotype. Increased mitochondrial biogenesis and maintenance of mitochondrial DNA have been observed in conditions of oxidative stress.^30^ Upon stem cell differentiation into motor neurons, an increase in mitobiogenesis was also observed that correlated with a transition from glycolysis to OXPHOS.^31^ Another potential explanation for the shift observed following mitochondrial transplantation may be a result of compensatory fusion of mitochondria that would alter the number of mitochondria impacting metabolic functions.^32^ As an example, in heart failure, despite the low energy state, a subpopulation of mitochondria in failing hearts remains active.^33^

## 3 Conclusion

Herein, we developed a strategy to polymerically functionalize isolated mitochondria for purposes of transplantation into cells and tissues to alter dynamics of energy handling such as substrate selection and efficiency of electron transport. A Dextran‐TPP polymer conjugate comprehensively coated isolated mitochondria, with the coating shown to protect mitochondrial respiratory function. Our results highlight efficient internalization of dextran‐coated mitochondria into breast cancer and cardiac cell lines, with an approximate 3‐fold increase compared with uncoated mitochondria. Last, mitochondrial transplantation into both cancer and cardiac cell lines resulted in a significant shift in the bioenergetic phenotype from a glycolytic to oxidative state. Our findings represent the first demonstration of polymer functionalization of mitochondria for purposes of cellular transplantation, opening potential avenues for translation of this approach for treatment of diseases characterized by metabolic impairment. In light of our promising findings, future studies will focus on in vivo evaluation of this approach in relevant models of cancer, heart failure, and neurodegenerative disease.

### 4 Experimental Section

*Dextran Polymer Conjugation and Characterization: N*,*N*′‐dicyclohexylcarbodiimide (Sigma‐Aldrich, St. Louis, MO, USA), 4‐(Dimethylamino) pyridine (Sigma‐Aldrich), and (5‐Carboxypentyl) triphenylphosphonium bromide (TPP‐COOH, Alfa Aesar, Lancashire, UK) were dissolved in anhydrous dimethyl sulfoxide (DMSO) and stirred for 5 min. The resulting mixture was added to DMSO solution containing dextran derived from *Leuconostoc mesenteroides* (*M*_w_ 150 k Da, Sigma‐Aldrich) and left stirring overnight. The solution underwent purification dialysis against water for 3 d using Slide‐A‐Lyzer MINI Dialysis Devices (3.5 K MWCO, Fisher Scientific, Waltham, MA, USA), after which functional Dextran‐TPP was obtained through lyophilization. Conjugation of TPP to dextran was confirmed by ^1^H NMR using a Varian 400 MHz NMR spectrometer (Santa Clara, CA, USA). Deuterated DMSO (Sigma‐Aldrich) was used as solvent. Grafted TPP amount on each dextran chain was calculated from the ratio of the characteristic peak area between TPP and dextran.

To visualize polymer coating, fluorescein 5(6)‐isothiocyanate (FITC, Sigma‐Aldrich) with λ_ex_ = 495 nm and λ_em_ = 525 nm was conjugated onto Dextran‐TPP polymer by mixing Dextran‐TPP and FITC (molar ratio of 1:3 Dextran‐TPP:FITC) in DMSO for 4 h in the dark, followed by dialysis of the mixture against water to remove residual solvent and unconjugated FITC, after which, Dextran‐TPP:FITC was lyophilized.

*Cell Culture*: The human triple negative breast adenocarcinoma cell line MDA‐MB‐231 (ATCC, Manassas, VA) was cultured with Leibovitz's L‐15 medium (ATCC), supplemented with 10% (v:v) fetal bovine serum (ThermoFisher Scientific) and 1% (v:v) penicillin/streptomycin (ThermoFisher Scientific) in a humidified incubator with atmospheric air at 37 °C. TNBC cells SUM‐159PT (Asterand, Detroit, MI, USA), mouse fibroblast L929 cells (Sigma‐Aldrich), BD1X rat heart myoblast H9c2 cells (ATCC), and human cervical carcinoma HeLa cells (ATCC) were all maintained in DMEM‐Dulbecco's Modified Eagle Medium (ThermoFisher Scientific) supplemented with 10% (v:v) fetal bovine serum and 1% (v:v) penicillin/streptomycin in a humidified incubator at 37 °C with 5% CO_2_.

*Mitochondrial Isolation from Cells*: All chemicals used in mitochondrial isolation were obtained from Sigma‐Aldrich. HeLa cells were washed with ice‐cold biopsy preservation solution (BIOPS) buffer (2.8 × 10^−3^ m CaK_2_egtazic acid (EGTA), 7.2 × 10^−3^ m K_2_EGTA, 5.7 × 10^−3^ m Na_2_ATP, 6.6 × 10^−3^ m MgCl_2_·6H_2_O, 20 × 10^−3^ m taurine, 15 × 10^−3^ m Na_2_phospho‐creatine, 20 × 10^−3^ m imidazole, 0.5 × 10^−3^ m dithiothreitol, 50 × 10^−3^ m 2‐(*N*‐morpholino)ethanesulfonic acid (MES), pH 7.1), detached with a cell scraper, and transferred to 1.5 mL Eppendorf tubes. Following gentle homogenization of cells in buffer A (220 × 10^−3^ m mannitol, 70 × 10^−3^ m sucrose, 5 × 10^−3^ m 3‐(*N*‐morpholino)propanesulfonic acid (MOPS), pH 7.4), buffer B (2 × 10^−3^ m EGTA, 0.2% free fatty acid‐free bovine serum albumin (BSA) in buffer A) was added to a volume of 1.5 mL, and then centrifuged at 800 rpm for 10 min at 4 °C. The supernatant was transferred to another tube filled with cold buffer B and centrifuged at 12 000 rpm for 5 min at 4 °C. The pellet was washed twice in ice‐cold buffer B and one time in cold buffer A, resuspended and centrifuged at 12 000 rpm for 5 min at 4 °C. Pellets were resuspended in 30 µL of cold buffer E (0.5 × 10^−3^ m of EGTA in buffer A). Mitochondrial number was normalized with protein concentration measured using a bicinchoninic acid assay (BCA) protein assay (Bio‐Rad, Hercules, CA, USA).

*Mitochondrial Isolation from Mice Tissue*: All animal studies were approved by the Institutional Animal Care and Use Committee of the Houston Methodist Research Institute. C57BL/6J mice (Jackson Laboratory, Bar Harbor, ME, USA), aged 7–9 weeks, were used for isolation of mitochondria from liver and skeletal muscle. For liver mitochondrial isolation, mice were sacrificed, livers quickly excised and washed with ice‐cold BIOPS buffer. Tissue was then minced in a petri dish with minimal ice‐cold buffer A. Tissue was transferred to 1.5 mL Eppendorf tubes and homogenized with a hand‐held pestle grinder in buffer A and centrifuged at 800 rpm for 10 min at 4 °C. The supernatant was transferred to another tube and centrifuged at 12 000 rpm for 5 min. The pellet was resuspended in cold buffer B and centrifuged at 12 000 rpm for 5 min. The resulting pellet was rinsed with buffer A and centrifuged at 12 000 rpm for 5 min, after which the pellet was resuspended in 30 µL of cold buffer E.

For mitochondrial isolation from muscle, skeletal muscle tissue (250–500 mg) was minced into small pieces in minimal ice‐cold BIOPS buffer, and then transferred to a dounce homogenizer vessel and incubated with 1 mL of ice‐cold fresh proteinase medium (2 mg mL^−1^ proteinase Subtilisin A in ATP medium [100 × 10^−3^ m KCl, 50 × 10^−3^ m Tris, 5 × 10^−3^ m MgSO_4_, 1 × 10^−3^ m EDTA, 1 × 10^−3^ m ATP, 0.05% BSA, pH 7.4]), mixed well and left to settle for 3 min. The supernatant was removed and the pellet resuspended in 6 mL ice‐cold ATP medium, followed by tissue homogenization using a dounce homogenizer on ice. The homogenate was then transferred to a 15 mL centrifuge tube, and centrifuged at 1700 rpm for 5 min at 4 °C. The supernatant was decanted into another 15 mL centrifuge tube and centrifuged at 3700 rpm for 20 min at 4 °C. The pellet was then suspended in 1 mL KCl medium (100 × 10^−3^ m KCl, 50 × 10^−3^ m Tris, 5 × 10^−3^ m MgSO_4_, 1 × 10^−3^ m EDTA, pH 7.4) transferred to a 1.5 mL Eppendorf tube, and centrifuged at 6800 rpm for 10 min at 4 °C. The residual pellet was resuspended in KCl medium and centrifuged. The final pellet was resuspended in 30 µL buffer E.

*CM Isolation from Adult Mice*: CMs were isolated from 8 to 10 weeks old C57BL/6J mice (Jackson Laboratory) by enzymatic digestion with a Langendorff perfusion system (Hugo Sachs Elektronik, Germany) followed by calcium reintroduction. The collagenase cocktail isolation perfusion buffer contained 0.15 mg mL^−1^ Liberase (Roche LifeScience, Indianapolis, IN, USA).

*Mitochondrial Coating with Dextran‐TPP*: Dextran‐TPP in buffer E was mixed with the pellet of isolated mitochondria at concentrations ranging from 3 to 6 mg mL^−1^, corresponding to weight ratios of polymer to mitochondria protein (1.4:1 to 2.9:1), and left shaking for 20 min at 4 °C. After incubation for another 20 min at 4 °C, coated mitochondria were centrifuged and washed 2× with buffer E to remove excess Dextran‐TPP. Uncoated mitochondria, serving as a control, underwent the same process. Mitochondria coated with fluorescent polymer were obtained using the same process with FITC‐labeled Dextran‐TPP.

*Cellular Uptake of Mitochondria*: H9c2 rat heart myoblast cells and L929 mouse fibroblast cells were cultured in 8‐well chamber slides overnight at a density of 1.6 × 10^4^ cells and 2.0 × 10^4^ cells per well, respectively. Cells were treated with uncoated or fluorescently coated mitochondria obtained from HeLa cells at a dose of 3 µg mitochondrial protein per 1.5 × 10^4^ cells. After 4 and 24 h, immunofluorescent detection of HeLa‐derived mitochondria in cells was performed by incubation with anti‐human mitochondria monoclonal antibody MTC02 (Abcam, Cambridge, MA) in 1% BSA solution overnight at 4 °C after fixing, permeabilizing, and blocking with 2% BSA in phosphate buffered saline (PBS). Anti‐Mouse IgG H&L (Cy3) preadsorbed (Abcam) was applied as a secondary antibody to visualize HeLa‐derived mitochondria in H9c2 rat and L929 mouse cells. F‐actin was stained with Alexa Fluor 647 Phalloidin (ThermoFisher Scientific). Slides were mounted with 4′,6‐diamidino‐2‐phenylindole (DAPI) medium onto a coverslip and examined using confocal microscopy.

Mitochondrial uptake was also examined in cancer cell lines. MDA‐MB‐231 cells and SUM‐159PT cells were seeded in 8‐well chamber slides overnight at a density of 2 × 10^4^ cells and 1.2 × 10^4^ cells per well, respectively. Cells were then incubated with fluorescently coated mitochondria obtained from mouse liver at a dose of 3 µg mitochondrial protein per 1.5 × 10^4^ cells. At predetermined time points (0.5, 4, and 24 h), cells were washed 2× with PBS, fixed with 4% paraformaldehyde at room temperature (RT) for 20 min, and permeabilized with 0.1% Triton X‐100 for 5 min. F‐actin was stained with Alexa Fluor 568 Phalloidin (ThermoFisher Scientific). Cells were mounted with DAPI medium before imaging using a Nikon A1 Confocal Imaging System (Melville, NY, USA).

*Mitochondrial Functional Analysis*: Mitochondrial respiratory function was assessed with Oroboros high‐resolution respirometry (Innsbruck, Austria) using coated and uncoated mitochondria. Mitochondria were suspended in MiR05 medium (0.5 × 10^−3^ m EGTA, 3 × 10^−3^ m MgCl_2_·6H_2_O, 60 × 10^−3^ m K‐lactobionate, 2 × 10^−3^ m taurine, 10 × 10^−3^ m KH_2_PO_4_, 20 × 10^−3^ m HEPES, 110 × 10^−3^ m sucrose, 1 g L^−1^ fatty acid‐free bovine serum albumin, pH 7.1) in Oroboros chambers with final concentration ≈0.1 mg mL^−1^ mitochondrial protein. The substrates pyruvate‐malate (PM, 5 × 10^−3^ m each), ADP (4 × 10^−3^ m), and oligomycin (5 × 10^−6^ m) were sequentially added to measure OXPHOS, LEAK, and OXPHOS capacity. RCR, indices of coupling between respiration and OXPHOS, were calculated as the ratio of state 3 (ADP‐supported respiration) to oligomycin state 4 (ATP‐synthase‐independent respiration after oligomycin addition). All readings were normalized for mitochondrial protein content as determined by BCA protein assay (Bio‐Rad).

*Extracellular Flux Analysis*: Cells were plated (3 × 10^4^ cells/well) in 24‐well Seahorse XF24 cell culture microplates 6 h prior to incubation with mitochondria. In the case of isolated CMs, cells were cultured onto laminin‐coated seahorse cell plates (1 × 10^4^ cells/well) with 5% fetal bovine serum overnight prior to incubation with mitochondria. At a time point of 24 h after incubation, plates were washed twice with seahorse assay medium (Seahorse base medium containing 25 × 10^−3^ m D‐glucose, 1 × 10^−3^ m sodium pyruvate, and 1 × 10^−3^ m l‐glutamine), the assay medium changed and maintained at 37 °C in a non‐CO_2_ incubator for 30 min. After baseline measurements, the medium was injected sequentially with: (1) oligomycin (1 × 10^−6^ m); (2) FCCP (1 × 10^−6^ m); and (3) rotenone (0.5 × 10^−6^ m) plus antimycin A (0.5 × 10^−6^ m). OCR and ECAR were measured using the Seahorse XF24 Analyzer (Agilent, Santa Clara, CA), as recommended by the manufacturer for Mito Stress Test Kit (Agilent Technologies, 13015–100). OCR and ECAR measurements were normalized to cell number in each well.

*Statistical Analyses*: GraphPad Prism software (version 7.00 for windows, GraphPad Software, La Jolla, CA, USA, www.graphpad.com) was used for statistical analysis unless otherwise stated. Results were expressed as mean ± SEM. Student's t‐test was used to assess differences between means of two independent data sets. One‐way ANOVA followed by Tukey's multiple comparison tests was used for differences in oxygen flux and extracellular acidification rate. A *p* value < 0.05 was considered significant.

## Conflict of Interest

The authors declare no conflict of interest.

## Supporting information

Supplementary

## References

[1] M. G. Vander Heiden , L. C. Cantley , C. B. Thompson , Science 2009, 324, 1029. 19460998 10.1126/science.1160809 PMC2849637

[2] G. J. Yoshida , J. Exp. Clin. Cancer Res. 2015, 34, 111. 26445347 10.1186/s13046-015-0221-y PMC4595070

[3] a) S. Neubauer , M. Horn , M. Cramer , K. Harre , J. B. Newell , W. Peters , T. Pabst , G. Ertl , D. Hahn , J. S. Ingwall , K. Kochsiek , Circulation 1997, 96, 2190; 9337189 10.1161/01.cir.96.7.2190

[4] K. Winkler‐Stuck , E. Kirches , C. Mawrin , K. Dietzmann , H. Lins , C. W. Wallesch , W. S. Kunz , F. R. Wiedemann , J. Neural Transm. 2005, 112, 499. 15340872 10.1007/s00702-004-0195-y

[5] A. Federico , E. Cardaioli , P. Da Pozzo , P. Formichi , G. N. Gallus , E. Radi , J. Neurol. Sci. 2012, 322, 254. 22669122 10.1016/j.jns.2012.05.030

[6] a) T. A. Ajith , T. G. Jayakumar , World J. Cardiol. 2014, 6, 1091; 25349653 10.4330/wjc.v6.i10.1091 PMC4209435

[7] A. M. Falchi , V. Sogos , F. Saba , M. Piras , T. Congiu , M. Piludu , Histochem. Cell Biol. 2013, 139, 221. 23108569 10.1007/s00418-012-1045-x

[8] K. Hayakawa , E. Esposito , X. Wang , Y. Terasaki , Y. Liu , C. Xing , X. Ji , E. H. Lo , Nature 2016, 535, 551. 27466127 10.1038/nature18928 PMC4968589

[9] M. V. Jackson , T. J. Morrison , D. F. Doherty , D. F. Mcauley , M. A. Matthay , A. Kissenpfennig , C. M. O'Kane , A. D. Krasnodembskaya , Stem Cells 2016, 34, 2210. 27059413 10.1002/stem.2372 PMC4982045

[10] X. Wang , H. H. Gerdes , Cell Death Differ. 2015, 22, 1181. 25571977 10.1038/cdd.2014.211 PMC4572865

[11] J. L. Spees , S. D. Olson , M. J. Whitney , D. J. Prockop , Proc. Natl. Acad. Sci. USA 2006, 103, 1283. 16432190 10.1073/pnas.0510511103 PMC1345715

[12] M. N. Islam , S. R. Das , M. T. Emin , M. Wei , L. Sun , K. Westphalen , D. J. Rowlands , S. K. Quadri , S. Bhattacharya , J. Bhattacharya , Nat. Med. 2012, 18, 759. 22504485 10.1038/nm.2736 PMC3727429

[13] Y. Zhao , Z. Jiang , E. Delgado , H. Li , H. Zhou , W. Hu , M. Perez‐Basterrechea , A. Janostakova , Q. Tan , J. Wang , M. Mao , Z. Yin , Y. Zhang , Y. Li , Q. Li , J. Zhou , Y. Li , E. Martinez Revuelta , J. Maria Garcia‐Gala , H. Wang , S. Perez‐Lopez , M. Alvarez‐Viejo , E. Menendez , T. Moss , E. Guindi , J. Otero , Stem Cells Transl. Med. 2017, 6, 1684. 28685960 10.1002/sctm.17-0078 PMC5689778

[14] E. Blanco , H. Shen , M. Ferrari , Nat. Biotechnol. 2015, 33, 941. 26348965 10.1038/nbt.3330 PMC4978509

[15] M. Ferrari , Nat. Rev. Cancer 2005, 5, 161. 15738981 10.1038/nrc1566

[16] A. K. A. Silva , D. Letourneur , C. Chauvierre , Theranostics 2014, 4, 579. 24723980 10.7150/thno.7688 PMC3982129

[17] C. Tassa , S. Y. Shaw , R. Weissleder , Acc. Chem. Res. 2011, 44, 842. 21661727 10.1021/ar200084x PMC3182289

[18] J. M. Harris , R. B. Chess , Nat. Rev. Drug Discovery 2003, 2, 214. 12612647 10.1038/nrd1033

[19] Z. Liu , Y. Jiao , Y. Wang , C. Zhou , Z. Zhang , Adv. Drug Delivery Rev. 2008, 60, 1650. 10.1016/j.addr.2008.09.001 18848591

[20] S. Biswas , N. S. Dodwadkar , A. Piroyan , V. P. Torchilin , Biomaterials 2012, 33, 4773. 22469294 10.1016/j.biomaterials.2012.03.032 PMC3725283

[21] R. A. Smith , C. M. Porteous , A. M. Gane , M. P. Murphy , Proc. Natl. Acad. Sci. USA 2003, 100, 5407. 12697897 10.1073/pnas.0931245100 PMC154358

[22] a) J. L. Barger , M. D. Brand , B. M. Barnes , B. B. Boyer , Am. J. Physiol.: Regul., Integr. Comp. Physiol. 2003, 284, R1306; 12676751 10.1152/ajpregu.00579.2002

[23] S. Salatin , S. Maleki Dizaj , A. Yari Khosroushahi , Cell Biol. Int. 2015, 39, 881. 25790433 10.1002/cbin.10459

[24] Z. Hu , Y. Sim , O. L. Kon , W. H. Ng , A. J. Ribeiro , M. J. Ramos , P. A. Fernandes , R. Ganguly , B. Xing , F. Garcia , E. K. Yeow , Bioconjugate Chem. 2017, 28, 590. 10.1021/acs.bioconjchem.6b00682 28049291

[25] a) S. Ly , D. M. Navaroli , M. C. Didiot , J. Cardia , L. Pandarinathan , J. F. Alterman , K. Fogarty , C. Standley , L. M. Lifshitz , K. D. Bellve , M. Prot , D. Echeverria , S. Corvera , A. Khvorova , Nucleic Acids Res. 2017, 45, 15; 27899655 10.1093/nar/gkw1005 PMC5224471

[26] a) A. K. Kaza , I. Wamala , I. Friehs , J. D. Kuebler , R. H. Rathod , I. Berra , M. Ericsson , R. Yao , J. K. Thedsanamoorthy , D. Zurakowski , S. Levitsky , P. J. Del Nido , D. B. Cowan , J. D. McCully , J. Thorac. Cardiovasc. Surg. 2017, 153, 934; 27938904 10.1016/j.jtcvs.2016.10.077

[27] a) M. M. Anastacio , E. M. Kanter , C. M. Makepeace , A. D. Keith , H. Zhang , R. B. Schuessler , C. G. Nichols , J. S. Lawton , Circulation 2013, 128, S130; 24030396 10.1161/CIRCULATIONAHA.112.000128 PMC3848320

[28] T. Nagoshi , M. Yoshimura , G. M. Rosano , G. D. Lopaschuk , S. Mochizuki , Curr. Pharm. Des. 2011, 17, 3846. 21933140 10.2174/138161211798357773 PMC3271354

[29] J. Zheng , Oncol. Lett. 2012, 4, 1151. 23226794 10.3892/ol.2012.928 PMC3506713

[30] H. C. Lee , Y. H. Wei , Int. J. Biochem. Cell Biol. 2005, 37, 822. 15694841 10.1016/j.biocel.2004.09.010

[31] L. C. O'Brien , P. M. Keeney , J. P. Bennett Jr. , Stem Cells Dev. 2015, 24, 1984. 25892363 10.1089/scd.2015.0076 PMC4545371

[32] R. J. Youle , A. M. van der Bliek , Science 2012, 337, 1062. 22936770 10.1126/science.1219855 PMC4762028

[33] A. M. Cordero‐Reyes , A. A. Gupte , K. A. Youker , M. Loebe , W. A. Hsueh , G. Torre‐Amione , H. Taegtmeyer , D. J. Hamilton , J. Mol. Cell. Cardiol. 2014, 68C, 98. 10.1016/j.yjmcc.2013.12.029 PMC3995348 24412531

