# Supplementary material for: Polymer Functionalization of Isolated Mitochondria for Cellular Transplantation and Metabolic Phenotype Alteration
Source: Adv Sci (Weinh). 2018 Jan 3;5(3):1700530. doi: 10.1002/advs.201700530 (PMC5867055; doi:10.1002/advs.201700530)
Supplement: Supplementary file 1 — Supplementary [file ADVS-5-1700530-s001.pdf]

Copyright WILEY-VCH Verlag GmbH & Co. KGaA, 69469 Weinheim, Germany, 2017.

ADVANCED SCIENCE
Open Access

Supporting Information

for *Adv. Sci.,* DOI: 10.1002/advs.201700530

Polymer Functionalization of Isolated Mitochondria for Cellular Transplantation and Metabolic Phenotype Alteration

Suhong Wu, Aijun Zhang, Shumin Li, Somik Chatterjee, Ruogu Qi, Victor Segura-Ibarra, Mauro Ferrari, Anisha Gupte, Elvin Blanco, and Dale J. Hamilton**

Supporting Information

Polymer functionalization of isolated mitochondria for cellular transplantation and metabolic phenotype alteration

Suhong Wu, Aijun Zhang, Shumin Li, Somik Chaterjee, Ruogu Qi, Victor Segura-Ibarra, Mauro Ferrari, Anisha Gupte, Elvin Blanco, Dale J. Hamilton

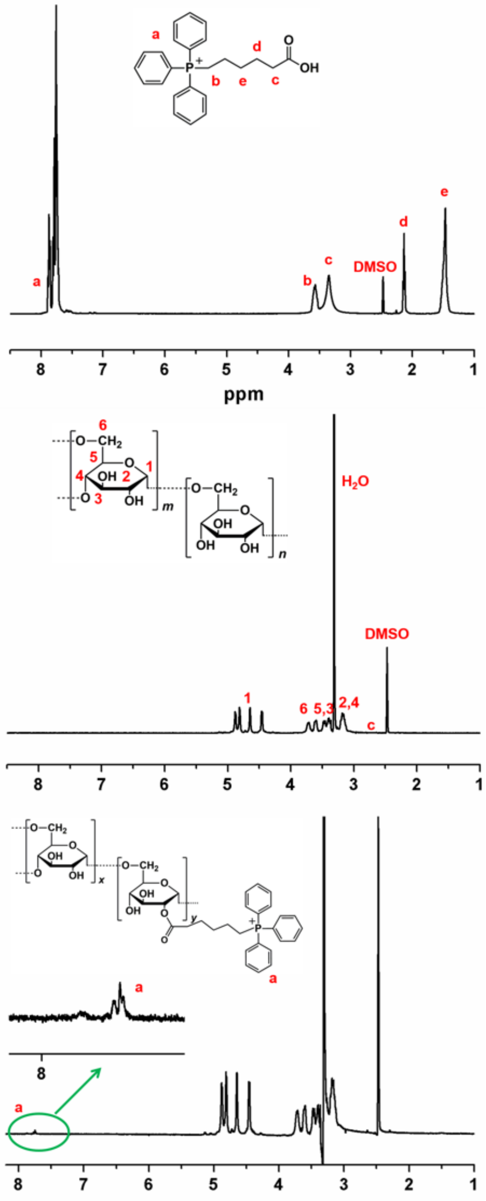

Figure S1. ^{1}H NMR spectrum of TPP (top), dextran (middle) and Dextran-TPP (bottom).

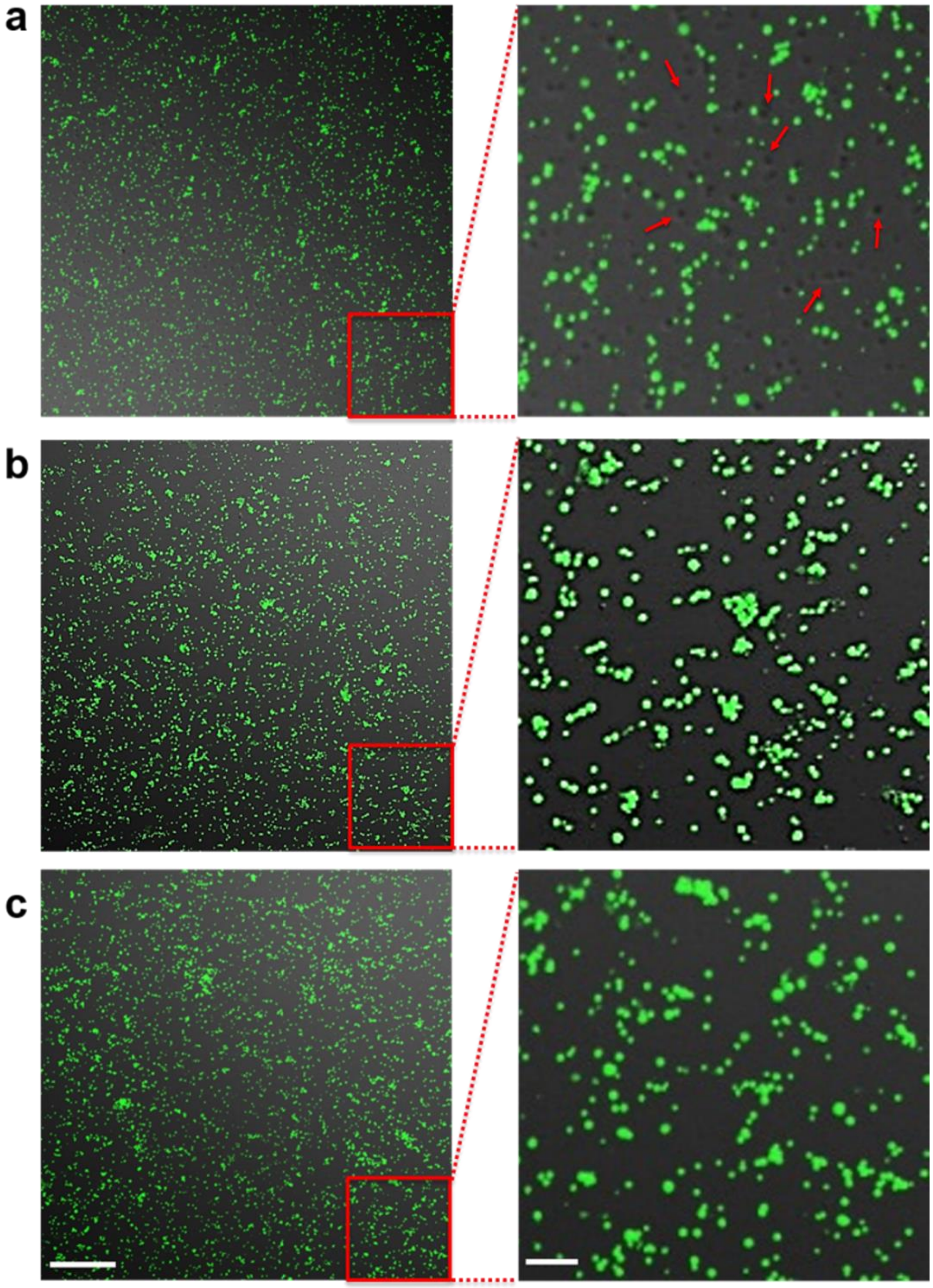

Figure S2. Confocal microscopy images of polymer coated mitochondria at various weight ratios of Dextran-TPP to mitochondrial protein. Mitochondria coated with a) 1.4x, b) 1.9x, and c) 2.9x polymer by weight. The scale bar in images on the left represents 50 µm. Images on the right represent magnified regions from left images. The scale bar in images on the right represents 10 µm. Red arrows denote uncovered mitochondria.

LEAK.

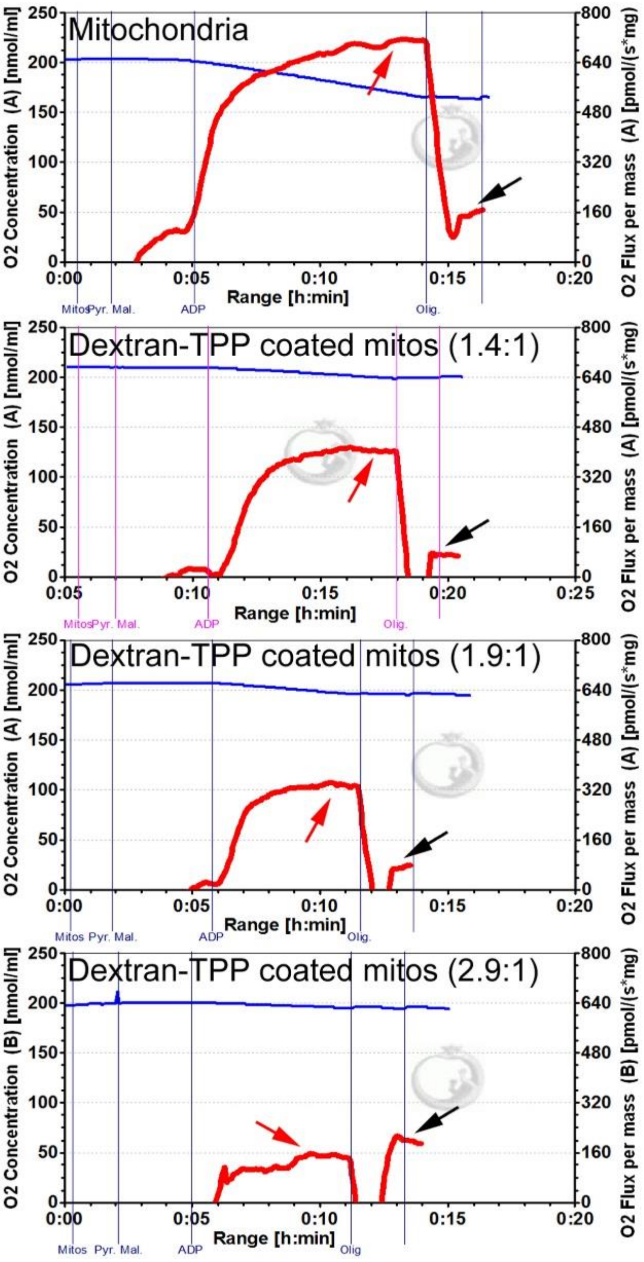

Figure S3. Oxygen consumption as a function of concentration of Dextran-TPP coating on mitochondria. Oxygen flux (right Y-axis) in response to sequential addition of pyruvate (Pyr, 5 mM) + malate (Mal, 2 mM), ADP (2 mM) followed by oligomycin (Olig, 4 µM). The red arrow indicated the state 3 rate (response to ADP), and the black arrow represents oligomycin state 4 LEAK.

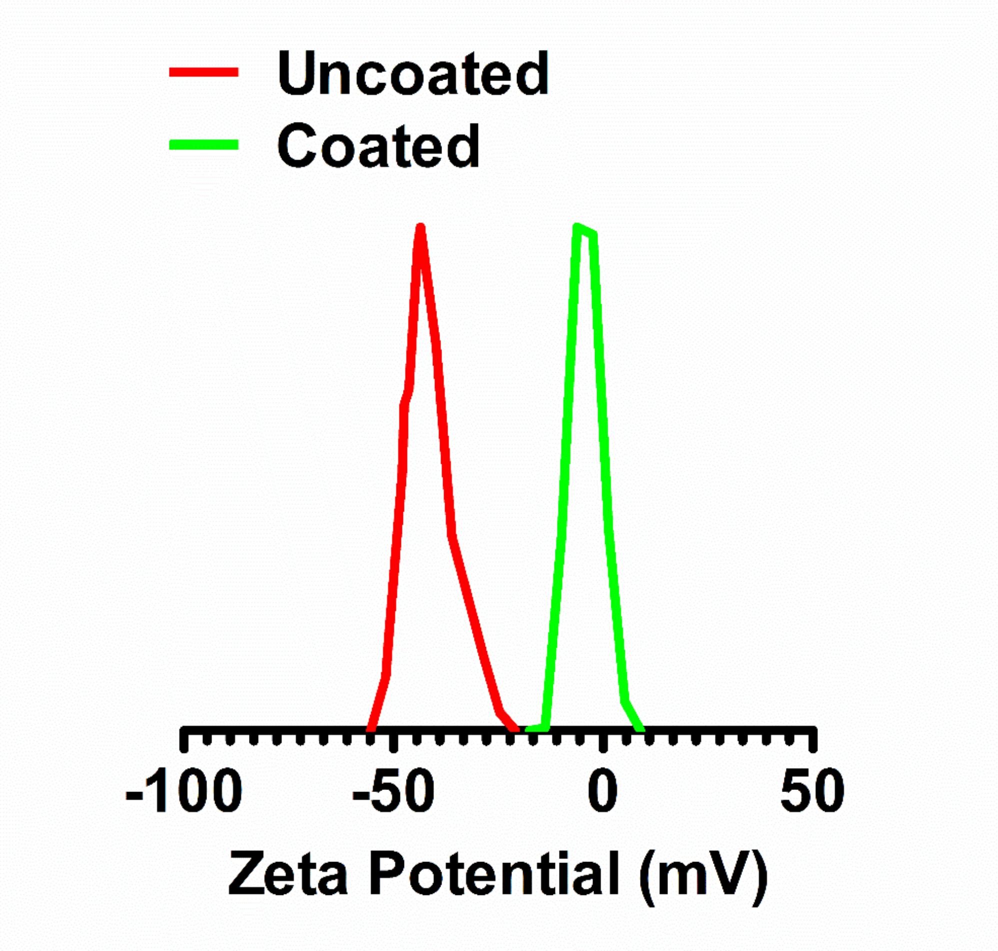

Figure S4. Zeta potential analysis of mitochondria prior to (red) and after polymer coating (green).

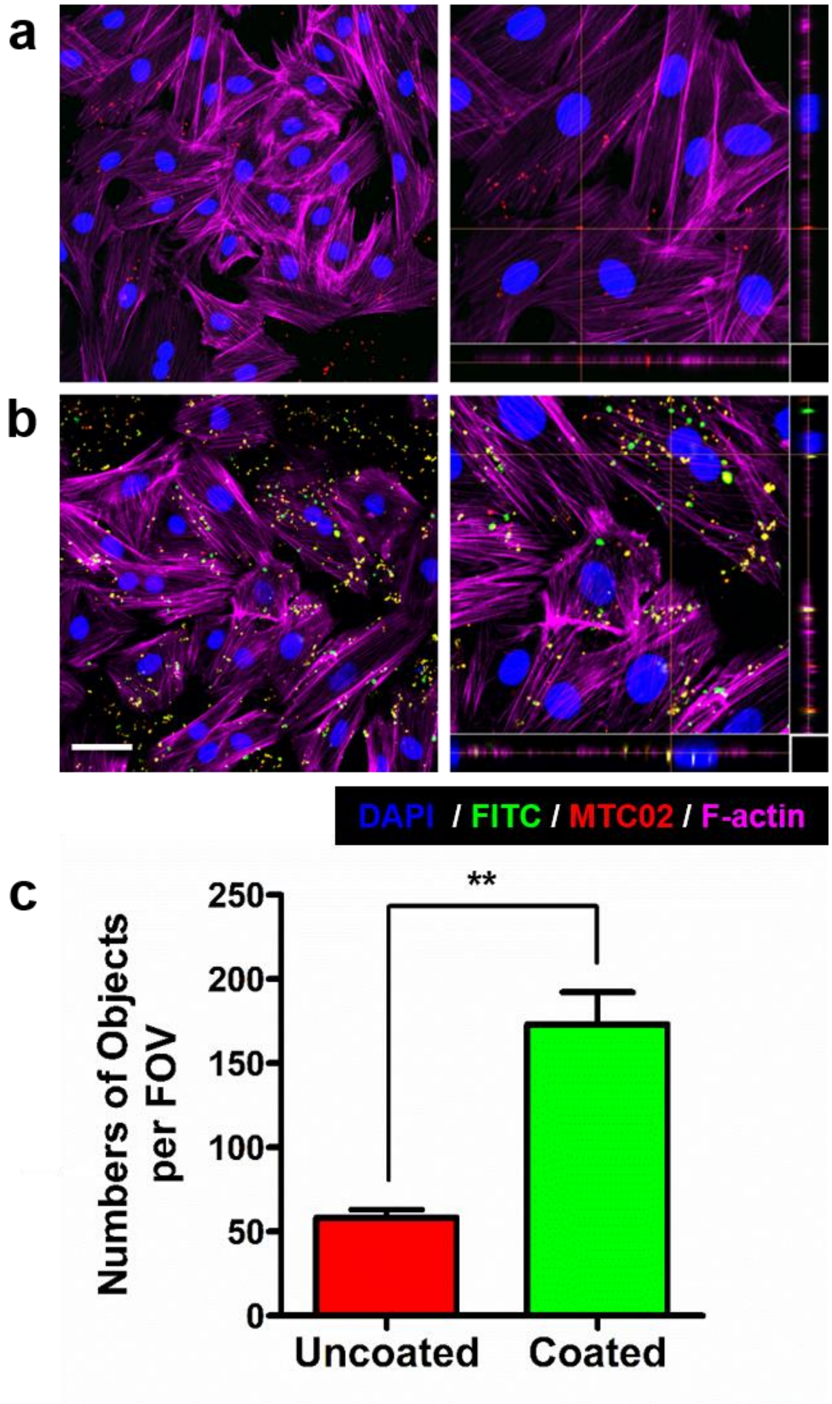

Figure S5. Uptake and intracellular localization of HeLa-derived mitochondria in H9c2 rat heart myoblast cells. Confocal microscopy images of H9c2 heart myoblast cells incubated for 4 h with (a) uncoated or (b) Dextran-TPP/FITC coated HeLa-derived mitochondria. Nuclei were stained with DAPI (blue) and mitochondria coated with Dextran-TPP/FITC (green). HeLa

mitochondria were detected with anti-human mitochondrial antibody (MTCO2) and anti-mouse IgG antibody (red). F-actin was stained with Alexa Fluor Phalloidin-647 (purple). Images represent low magnification (left) and 2D images (right), with panels below and to the right of 2D images highlighting mitochondrial internalization. The scale bar represents 50 µm. (c) Average number of internalized mitochondria, uncoated and Dextran-TPP/FITC coated, per field of view in H9c2 heart myoblast cells. Results Results represent mean ± SEM (**$P<0.01$ compared with uncoated mitos group).

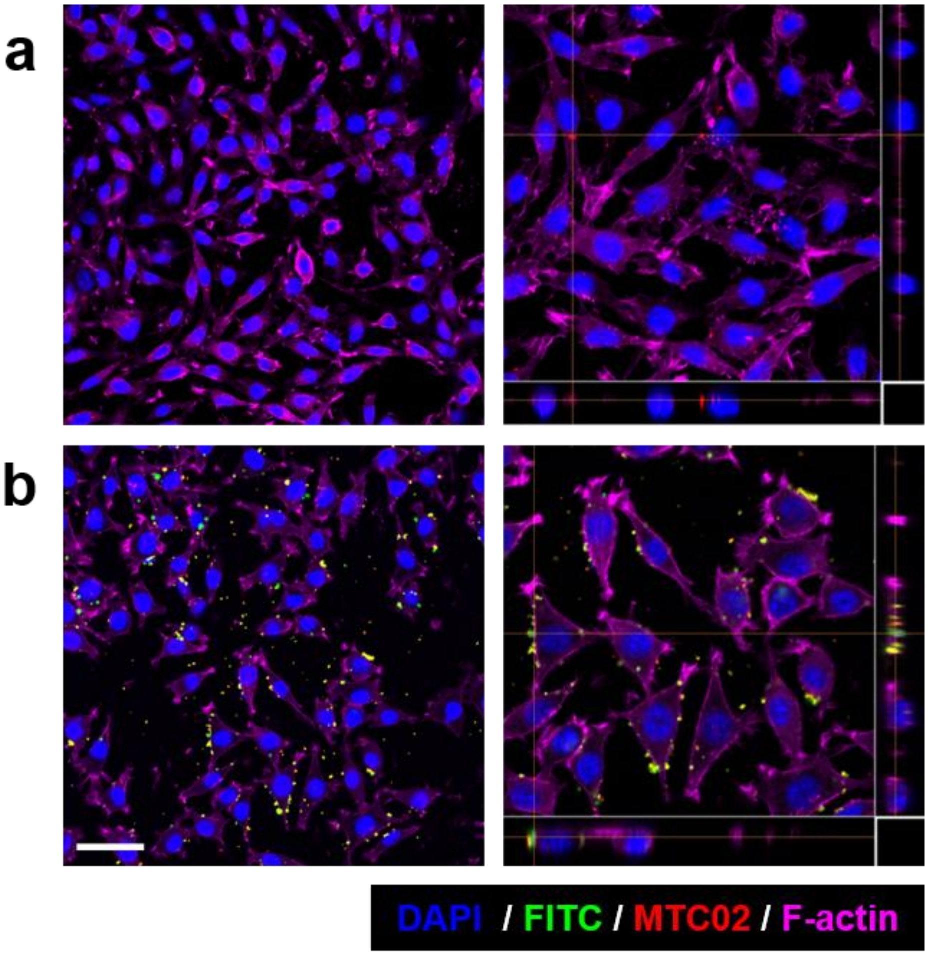

Figure S6. Uptake and intracellular localization of HeLa-derived mitochondria in L929 mouse fibroblast cells 4 h after administration. Confocal microscopy images of L929 mouse fibroblast cells incubated for 4 h with (a) uncoated or (b) Dextran-TPP/FITC coated HeLa-derived mitochondria. Nuclei were stained with DAPI (blue) and mitochondria coated with Dextran-TPP/FITC (green). HeLa mitochondria were detected with anti-human mitochondrial antibody (MTCO2) and anti-mouse IgG antibody (red). F-actin was stained with Alexa Fluor Phalloidin-647 (purple). Images represent low magnification (left) and 2D images (right), with panels below and to the right of the 2D images highlighting mitochondrial internalization. The scale bar represents 50 µm.

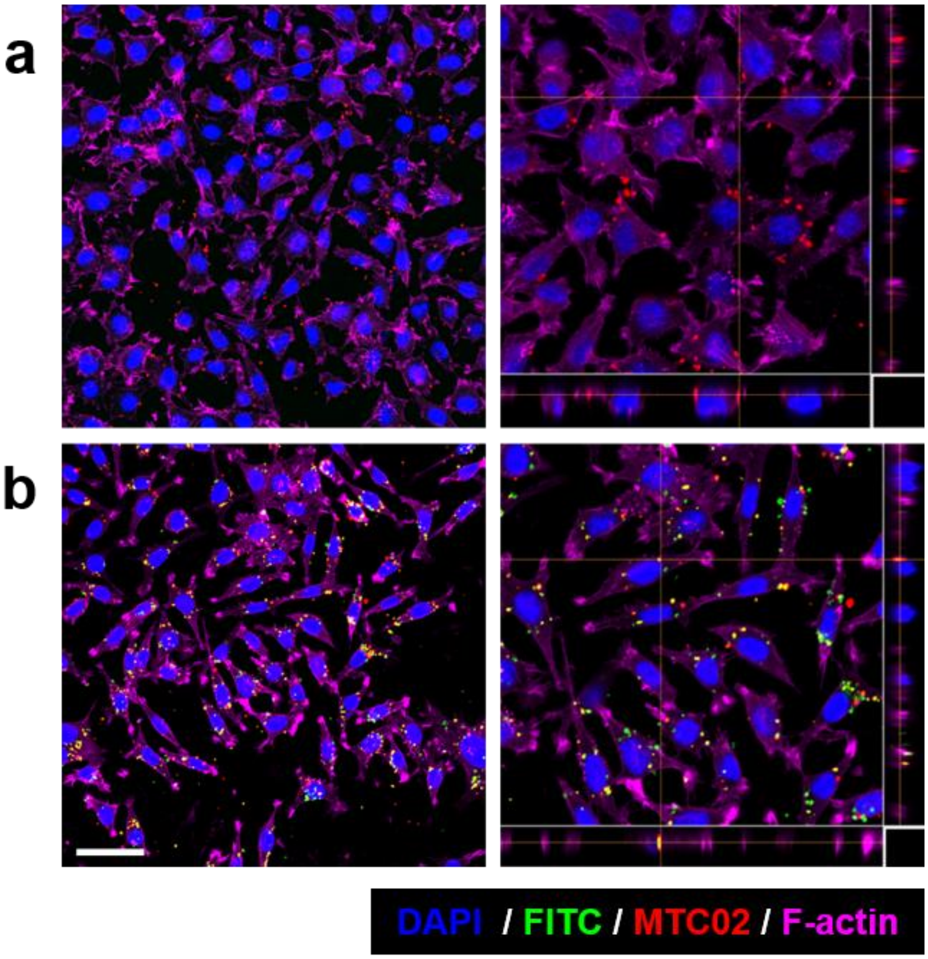

Figure S7. Uptake and intracellular localization of HeLa-derived mitochondria in L929 mouse fibroblast cells 24 h after administration. Confocal microscopy images of L929 mouse fibroblast cells incubated for 24 h with (a) uncoated or (b) Dextran-TPP/FITC coated HeLa-derived mitochondria. Nuclei were stained with DAPI (blue) and mitochondria coated with Dextran-TPP/FITC (green). HeLa mitochondria were detected with anti-human mitochondrial antibody (MTCO2) and anti-mouse IgG antibody (red). F-actin was stained with Alexa Fluor Phalloidin-647 (purple). Images represent low magnification (left) and 2D images (right), with panels below and to the right of the 2D images highlighting mitochondrial internalization. The scale bar represents 50 µm.

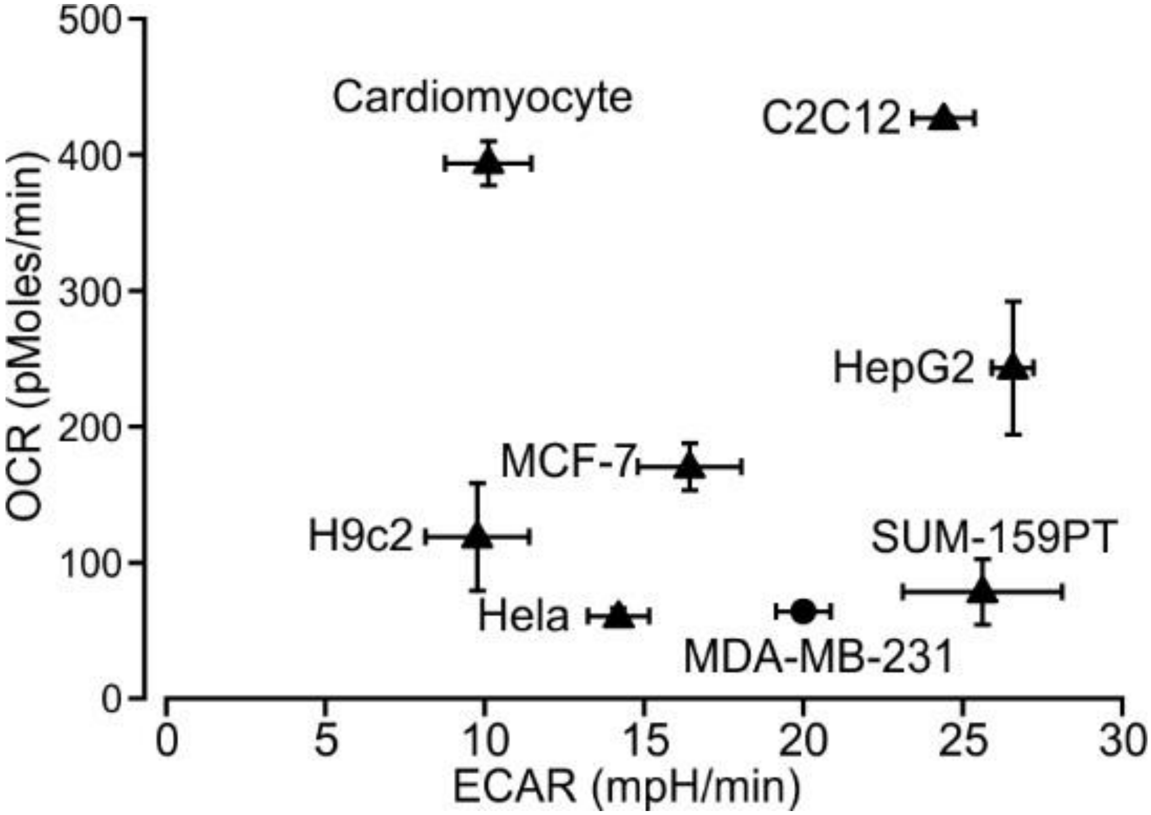

Figure S8. Energy metabolism phenotype of different cells. Basal oxygen consumption rate (OCR) as a function of extracellular acidification (ECAR) in 8 different cells and cell lines. Results represent mean ± SEM.

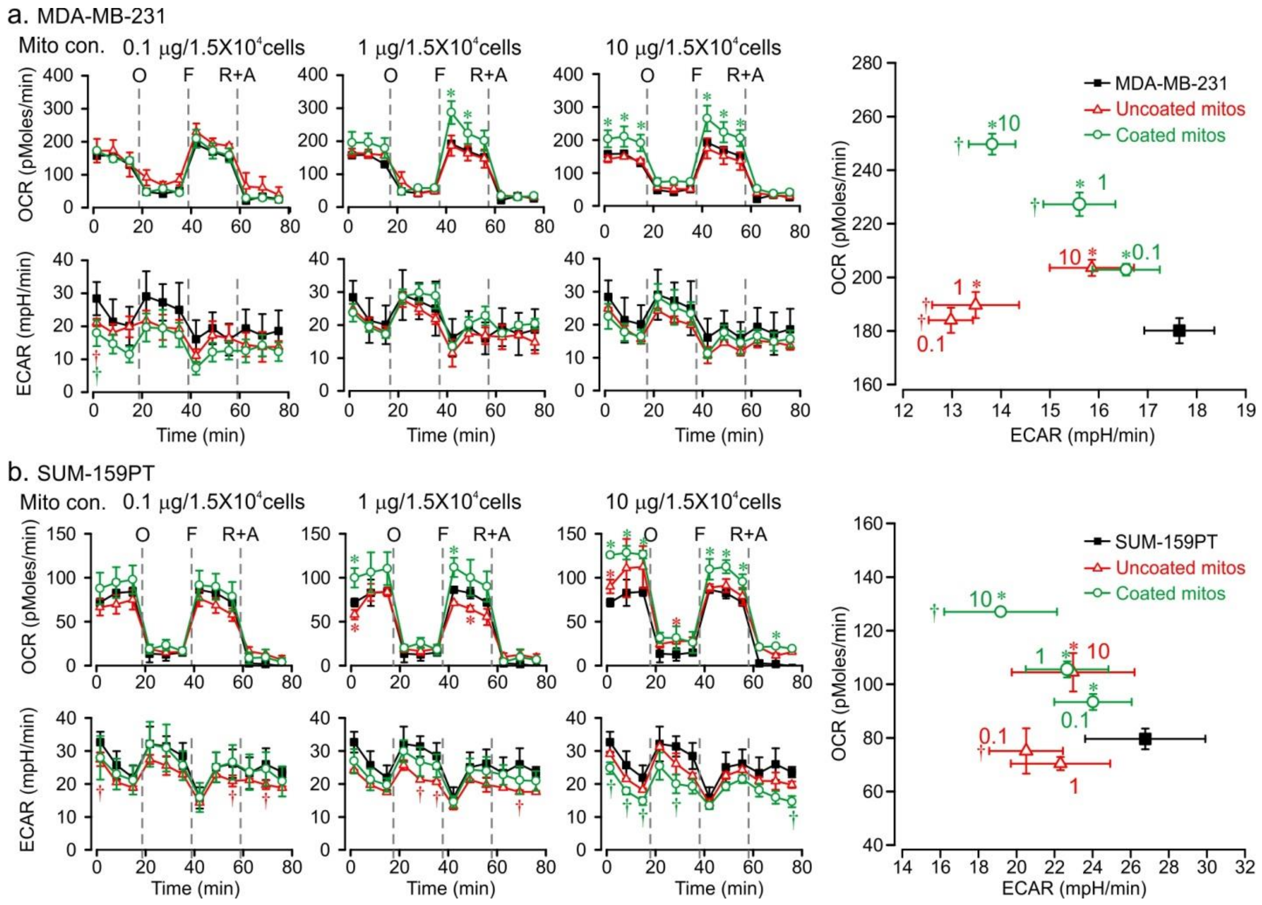

Figure S9. Dose effect on mitochondrial oxygen respiration following transplantation of Dextran-TPP coated mitochondria into human breast cancer cells. Oxygen consumption rate (OCR) and extracellular acidification (ECAR) dose response of MDA-MB-231 (a) and SUM-159PT (b) breast cancer cells 24 h after transplantation of uncoated (red) and Dextran-TPP coated (green) mouse liver-derived mitochondria at concentrations of 0.1, 1, and 10 µg mitochondrial protein per 1.5x10^{4} cells. OCR as a function of ECAR (right panels) is derived from basal measurements. Results represent mean ± SEM (*P<0.05 and †P<0.05 compared with OCR and ECAR, respectively, in non-treated cells). O: oligomycin; F: FCCP; R+A: rotenone + antimycin A.

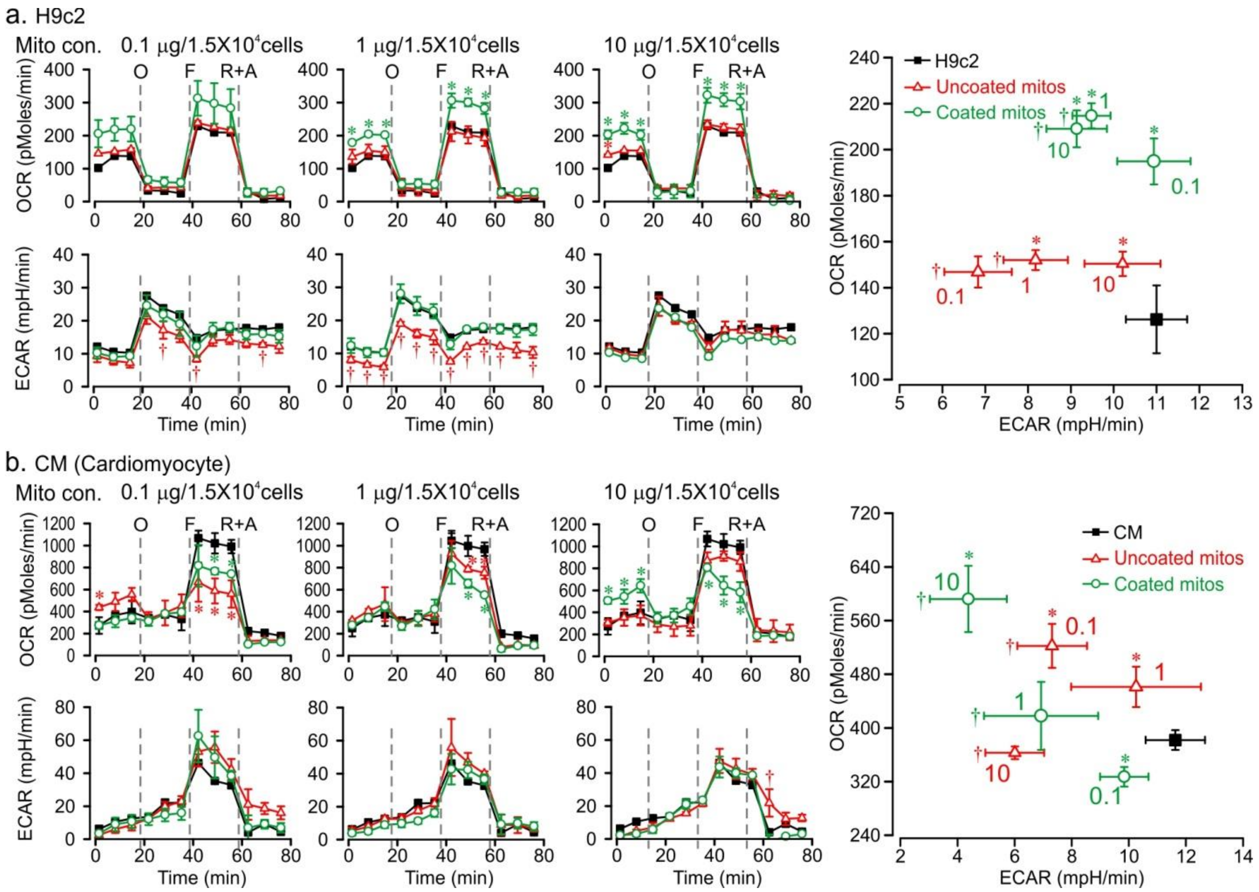

Figure S10. Dose effect on mitochondrial oxygen respiration following transplantation of Dextran-TPP coated mitochondria into cardiac cells. Oxygen consumption rate (OCR) and extracellular acidification (ECAR) dose response of H9c2 heart myoblast cells (a) and primary adult mouse cardiomyocytes (b) 24 h after transplantation of uncoated (red) and Dextran-TPP coated (green) mouse liver-derived mitochondria at concentrations of 0.1, 1, and 10 μg mitochondrial protein per $1.5x10^4$ cells. OCR as a function of ECAR (right panels) is derived from basal measurements. Results represent mean ± SEM (*P<0.05 and †P<0.05 compared with OCR and ECAR, respectively, in non-treated cells). O: oligomycin; F: FCCP; R+A: rotenone + antimycin A.
